# Low energy diets supplemented with lysophosphatidylcholine improve production performance and lipid metabolism in broilers

**DOI:** 10.3389/fvets.2026.1753429

**Published:** 2026-02-18

**Authors:** Rui Liu, Xiaochuan Liu, Yuqiang Wei, Wenjing Liu, Tao Li, Xuejun Yuan, Shuzhen Jiang, Weiren Yang, Ning Jiao

**Affiliations:** 1 Key Laboratory of Efficient Utilization of Non-Grain Feed Resources (Co-construction by Ministry and Province), Ministry of Agriculture and Rural Affairs, College of Animal Science and Technology, Shandong Agricultural University, Tai’an, China; 2 Linyi Zhengneng Biotechnology Co., Ltd., Linyi, China; 3 College of Life Sciences, Shandong Agricultural University, Tai’an, China

**Keywords:** carcass traits, growth performance, lipid metabolism, lysophosphatidylcholine emulsifiers, meat quality

## Abstract

**Introduction:**

The high-energy diets commonly used in broiler production pose challenges for efficient lipid digestion. Supplementing exogenous emulsifiers is an effective strategy to enhance the digestibility of dietary energy and improve overall fat utilization. This study aimed to investigate the effects of dietary exogenous emulsifier lysophosphatidylcholine (LPC) supplementation on broilers fed a reduced energy diet.

**Methods:**

A total of 432 1-day-old Arbor Acres (AA) broilers were randomly assigned to four dietary treatments: the control group (CON, basal diet), the negative control group (NC, basal diet with 60 kcal/kg metabolic energy reduction), and the NC diet supplemented with 500 or 750 mg/kg LPC groups. Each treatment had 6 replicates, with 18 birds per replicate for 42 days.

**Results and discussion:**

Compared with the NC group, the addition of 500 and 750 mg/kg LPC both increased final weight and average daily gain (ADG) while decreasing feed-to-gain at 1–42 days (*p* < 0.05). Additionally, LPC supplementation at 750 mg/kg significantly increased OM and EE digestibility compared with the NC group (*p* < 0.05). In addition, LPC supplementation at 750 mg/kg improved the carcass traits at 21 and 42 days (*p* < 0.05). Moreover, the improved meat quality from LPC supplementation was evidenced by increased intramuscular fat, reduced drip loss at 500 mg/kg, and optimized meat color (L, a, and b*) at 750 mg/kg (*p* < 0.05). Furthermore, 750 mg/kg LPC positively modulated serum biochemistry and hepatic lipid metabolism, as evidenced by reduced activities of alanine aminotransferase and aspartate aminotransferase, and lower levels of triglycerides, total cholesterol, and malondialdehyde (*p* < 0.05). In conclusion, low-energy diets supplemented with LPC at 750 mg/kg effectively improved production performance, meat quality, and health status by regulating hepatic lipid metabolism, which provides a theoretical basis for its application in the poultry industry.

## 1 Introduction

Dietary energy level is the most critical nutritional parameter for poultry; however, high-energy diets, while meeting nutritional requirements, may also induce adverse effects. On one hand, the accelerated rise in feed ingredient prices has substantially increased production costs in animal farming (1, 2). On the other hand, the immature digestive systems of poultry make them susceptible to lipid metabolism disorders when fed high-energy diets (3, 4). Therefore, identifying feed additives that enhance lipid utilization efficiency while conserving feed resources has become a research priority.

Emulsifiers are amphiphilic macromolecular surfactants capable of forming interfacial bridges between hydrophilic and lipophilic phases (5). Bontempo et al. emphasized the possible utilization of exogenous emulsifiers as additives in high-yielding chickens (6). Research indicates that emulsifiers, when used as feed additives, enhance the effective utilization of dietary energy by improving lipid metabolism (7, 8). Growing evidence confirms their efficacy in low-energy broiler diets. Specifically, emulsifier supplementation has been shown to compensate for energy reduction by enhancing overall performance, dry matter (DM) digestibility, and intestinal morphology parameters (9). A synergistic effect is observed when emulsifiers are combined with multi-enzymes, thereby supporting improved growth performance and nutrient digestibility in young broilers and mitigating the negative impacts of dietary energy restriction (10). Importantly, a dose–response relationship has been identified, with recommendations suggesting that supplementing the emulsifier at a rate of at least 250 mg/kg of feed for every 100-kcal reduction in metabolizable energy can effectively enhance broiler performance throughout all growth phases by improving lipid digestibility and intestinal health (9, 11). Luca Marchetti et al. demonstrated that supplementation with solidified glycerol polyethylene glycol ricinoleate and bi-distilled oleic acid in reduced-energy diets significantly enhanced growth performance in broilers by improving nutrient digestibility and optimizing hepatic fatty acid profile (12). As a hydrolysis product of phosphatidylcholine catalyzed by phospholipases, lysophosphatidylcholine (LPC) represents the smallest and structurally simplest known phospholipid, serving as both a critical precursor in eukaryotic phospholipid biosynthesis and a key intermediate in glycerophospholipid metabolism (13, 14).

LPC, an exceptional exogenous emulsifier, has been widely adopted in livestock and poultry production. Dietary LPC supplementation at 0.05 ~ 0.1% in low-energy diets improved feed efficiency and reduced production costs in laying hens by enhancing fat digestibility and upregulating CAT-1 and NPC1 genes to promote nutrient absorption (15). Similarly, dietary LPC supplementation demonstrated multifaceted benefits by enhancing growth performance and immune response in broilers fed corn/soybean meal-based diets, while demonstrating the efficacy of lower-cost recycled fat sources as viable alternatives to support growth and reduce feed costs (16, 17). Recently, a study revealed that LPC supplementation altered jejunal morphology by modulating genes enriched in innate immune signaling pathways and processes such as endodermal cell differentiation and dipeptide transport (18). The study indicated that LPC supplementation increased average body weight, reduced feed conversion ratio, and improved ileal dry matter, crude fat, and crude protein (CP) digestibility, while decreasing abdominal fat deposition in broilers (19). It has been documented that dietary 2% soy–lecithin supplementation could increase the percentage of thigh muscle fat and serum high-density lipoprotein (HDL) level, while decreasing cholesterol (TC) and low-density lipoprotein cholesterol (LDL) levels in broilers (20). Moreover, a study demonstrated that dietary supplementation with 0.5 g/kg of enzymatically modified lecithin enhanced the apparent metabolism of fatty acids and elevated the metabolizable energy in broilers (21). Supplementation of LPC in the diet can improve growth performance and nutrient ileal digestibility in broilers by enhancing the expression of fatty acid transporter genes and upregulating the expression of key genes in the lipid β-oxidation pathway (22, 23). Nutautaitė et al. demonstrated that dietary supplementation with LPC significantly enhanced body weight and average daily gain (ADG) in broilers chickens (24).

However, studies on dietary LPC supplementation to improve broiler health under low-energy diets remain limited. Therefore, this study was designed to investigate whether dietary LPC supplementation could improve growth performance, carcass traits, meat quality, and hepatic lipid metabolism in broilers fed low-energy diets, providing a theoretical basis for its precise application in poultry nutrition and new insights for emulsifier research.

## 2 Materials and methods

### 2.1 Ethics statement

The broilers used in the present experiment were cared for in accordance with the Chinese Guidelines for Animal Welfare and Experimental Protocols, and the experiment was approved by the Animal Care and Use Committee of Shandong Agricultural University (SDAUA-2021-081).

### 2.2 Animals, treatments, and management

This experiment selected 432 1-day-old Arbor Acres (AA) broilers, which were randomly divided into four treatments, with 6 replicates per treatment and 18 broilers per replicate for a duration of 42 days. Broilers in the control (CON) and negative control (NC) groups received the basal diet and the basal diet with 60 kcal/kg of energy, respectively. The treatment groups were fed the NC diet supplemented with LPC at 500 or 750 mg/kg.

The diets were formulated to meet the recommended nutritional requirements of broilers, as specified in the National Standard of the People’s Republic of China, “Compound Feed for Laying Chickens and Broilers” (GB/T 5916-2020). The diets of the CON and NC groups were formulated to meet the nutritional requirements of broilers in two distinct phases: the starter phase (days 1–21) and the grower-finisher phase (days 22–42). By adjusting the composition ratio of feed ingredients, the goal of reducing 60 kcal/kg is achieved while maintaining the levels of other ingredients unchanged (Table 1). Throughout the experimental period, all diets (for both phases) were provided in pellet form, and all feed was stored in a dry and well-ventilated area. The broilers were raised in three-layer overlapping cages and fed with pellet feed. A 23 L:1D lighting regimen of 20 lx was provided from days 0–7; thereafter, an 18 L:6D regimen of 10 lx was maintained for the remainder of the experimental period (17). The broilers were immunized and managed according to the conventional feeding management procedures.

**Table 1:** Ingredients and nutrient levels of the experimental diets (%, as-fed basis).

Items	Phase 1 (days 1–21)		Phase 2 (days 22–42)	
	CON	NC	CON	NC
Ingredients				
Corn	44.00	47.00	53.00	50.50
Soybean meal 43% CP	22.20	20.60	24.00	23.00
Wheat bran	8.00	8.00	8.00	10.00
Corn gluten meal	4.00	7.00	3.00	5.69
Cottonseed meal 46% CP	14.00	10.00	4.00	4.00
Feather meal	0.50	0.42	1.00	0.80
Sodium bicarbonate	1.80	1.80	1.50	1.50
Limestone	1.20	1.20	0.60	0.60
Duck oil	1.00	0.60	2.00	1.00
Lysine	0.50	0.52	0.25	0.27
Methionine	0.70	0.73	0.59	0.59
Threonine	0.10	0.13	0.06	0.05
Premix^a^	2.00	2.00	2.00	2.00
Total	100.00	100.00	100.00	100.00
Nutrient levels^b^				
Metabolic energy (kcal/kg)	2970.00	2910.00	3050.00	2990.00
Crude protein	22.75	22.75	19.54	19.50
Calcium	0.96	0.95	0.66	0.66
Available phosphorus	0.45	0.44	0.37	0.37
Methionine	1.01	1.02	0.86	0.86
Lysine	1.34	1.33	1.08	1.08
Threonine	0.85	0.85	0.72	0.72

a Premix was provided per kg of diet: VA 9,050 IU; VD_3_ 1,950 IU; VE 26 IU; VK_3_ 5 mg; VB_1_ 2.6 mg; VB_2_ 8.0 mg; VB_6_ 3.0 mg; VB_12_ 0.02 mg; choline chloride 500 mg; VB_5_ (calcium pantothenate) 15 mg; niacin 35 mg; biotin 0.20 mg; folic acid 1.20 mg; Mn (MnSO_4_·H_2_O) 60 mg; Fe (FeSO_4_·H_2_O) 80 mg; Zn (ZnSO_4_·H_2_O) 60 mg; Cu (CuSO_4_·5H_2_O) 8.5 mg; I (KIO_3_) 0.27 mg; Se (Na_2_SeO3) 0.20 mg.

b Nutrient levels were calculated values. CON, control group; NC, negative control group; CP, crude protein.

### 2.3 Slaughter and sample collection

At the end of the experiment, one broiler with average body weight from each replicate was selected for blood collection and slaughter. Briefly, blood samples were collected from the wing vein under fasting conditions, and then centrifuged at 1,000 g for 10 min to obtain serum using the laboratory centrifuge (Thermo Fisher Scientific, MA, United States), which was stored at −20 °C until further analysis. Finally, six broilers of each group were humanely euthanized by cervical dislocation. Liver tissues were collected and stored at −80 °C for further lipid metabolism analysis. In addition, breast muscle and leg muscle were collected and stored at 4 °C for further meat quality analysis.

### 2.4 Growth performance

Feed intake and body weight of broilers were accurately recorded in replicates at days 1, 21, and 42 of the experiment. These data were used to calculate the average daily gain (ADG), average daily feed intake (ADFI), and feed-to-gain (F/G) ratio.

### 2.5 Apparent nutrient digestibility

The metabolic experiment was conducted on day 39 ~ 41. The feces of all 18 broilers in each replicate were collected continuously for 39 ~ 41 days. The excrements were carefully cleaned of feathers and exfoliated dandruff, weighed, mixed in duplicate, and stored at −20 °C. Before chemical analysis, fecal samples were dried at 60 °C for 72 h, then ground and passed through a 1-mm screen (25). Apparent metabolizable energy of dry matter (DM), organic matter (OM), ether extract (EE), and crude protein (CP) was determined using the acid-insoluble ash (AIA) method (12). The DM, OM, EE, CP, and AIA contents in feed and feces were determined according to the method of the AOAC (26). Apparent nutrient digestibility rate was calculated by the following formula: Apparent nutrient digestibility (%) = (1 – [feed AIA content/fecal AIA content] × [fecal nutrient content/feed nutrient content]) × 100 (27). The apparent metabolizable energy (AME) values were calculated based on the direct measurement of gross energy (GE). The GE of the diets and excreta was measured using an oxygen bomb calorimeter (HACH COD-60A, HACH, China). The AME was then calculated using the standard formula: AME of the diet (MJ/kg DM) = (Feed intake × Diet GE – Fecal output × Fecal GE)/Feed intake (28).

### 2.6 Carcass traits

The broilers were slaughtered and weighed with the giblets removed to obtain the half-carcass weight, which was then removed the internal organs to get eviscerated to obtain the eviscerated carcass. In addition, abdominal fat, breast muscle, and leg muscle were collected and weighed. The carcass traits were assessed following the Nomenclature and Metric Statistical Methods of Poultry Production Performance (NY/T 823-2020).

### 2.7 Serum biochemical indices

The serum levels of triglycerides (TG), total cholesterol (TC), high-density lipoprotein (HDL), low density lipoprotein (LDL), total protein (TP), albumin (ALB), alkaline phosphatase (ALP), alanine aminotransferase (ALT), aspartate aminotransferase (AST), urea nitrogen (UREA) and uric acid (UA) were analyzed using a Cobus-Mirs-plus automatic biochemical analyzer (Roche Diagnostic System, Inc., United States) following standard procedures. In addition, the content of malondialdehyde (MDA) was measured according to the manufacturer’s instructions using a commercial kit (Nanjing Jiancheng Bioengineering Institute, Nanjing, China).

### 2.8 Meat quality

Drip loss was measured as described previously (29). Briefly, almost 90 g of meat was hung in a plastic bag using a string under the lid at 4 °C for 24 h, and the bag was kept out of contact with the meat. Drip loss was presented as a percentage of the drip amount relative to the initial weight. At 45 min and 24 h postmortem, the pH of each sample was measured using a pH Star 6.05 instrument (Matthäus, Bavaria, Germany). Concurrently, the color parameters at 45 min, including L* (lightness), a* (redness), and b* (yellowness) values, were determined using a Colorimeter (Minolta CR410, Tokyo, Japan). Before measurement, the colorimeter was calibrated using the white tile. In addition, cooking loss was measured by cooking meat in a water bath until the geometric center reached 70 °C (monitored in real-time with a calibrated thermocouple probe), maintained at this temperature for 20 min to ensure complete pasteurization and enzyme inactivation. After cooking, the samples were cooled to room temperature and then weighed. Cooking loss was presented as a percentage of the loss relative to the initial weight. Furthermore, 10 cylindrical samples (1.0 in diameter) were obtained from cutting the above meat parallel to the fiber orientation, and measured by cutting the sample vertically to the fiber axis using a muscle tenderness meter (C-LM3B, Tenovo, Harbin, China), at a speed of 300 mm/min to measure the shear force.

### 2.9 Hepatic lipid metabolism

Hepatic lipid metabolism was measured as described previously (7). The liver samples were taken by opening the abdominal cavity, accurately cutting liver tissue samples (about 1 cm^3^) from the same position of the left lobe of the liver, and using sterile forceps to rapidly transfer the liver tissue into a 1.5 mL freezing tube before immediately putting it into liquid nitrogen and finally placing it into a − 80 °C refrigerator for storage. For sample processing, 1.00 ± 0.01 g of liver tissue was accurately weighed into a 1.5-mL centrifuge tube. A 10% homogenate was prepared on ice at a ratio of 1:9 (tissue: saline), while another aliquot was homogenized in ice-cold anhydrous ethanol at the same ratio for lipid extraction. Homogenization was performed at 4,550 *g* for 3 min. The homogenates were centrifuged at 740 g for 10 min at 4 °C, and the supernatant was carefully collected. Finally, TC, TG, and MDA were measured according to the instructions of the kit (Nanjing Jiancheng Bio-engineering Institute, Nanjing, China), and the OD value was recorded using the microplate reader (multifunctional enzyme-linked immunosorbent assay reader Synergy 4, BioTek, Inc., United States).

### 2.10 Statistical analysis

Data normality and homogeneity of variance were checked using the Shapiro–Wilk test and Levene’s test, respectively. All data satisfied parametric assumptions. The test results were statistically analyzed via one-way analysis of variance (ANOVA) using SAS (version 9.4, SAS Institute, Cary, NC, United States), and Tukey’s method was used for multiple comparison test among the treatments. Data are expressed as means and standard error of the mean. Differences were considered statistically significant at *p* < 0.05.

## 3 Results

### 3.1 Growth performance

The effects of LPC supplementation on broiler growth performance are summarized in Table 2. At 1 ~ 21 days, broilers fed the diet with 500 mg/kg LPC exhibited a significantly higher ADFI than the NC group (*p* < 0.05). Furthermore, supplementation with 750 mg/kg LPC significantly enhanced both ADG and ADFI compared with both the NC and CON groups (*p* < 0.05), and resulted in a significantly higher final body weight than the NC group (*p* < 0.05).

**Table 2:** Effects of LPC supplementation on growth performance in broilers.

Items	CON	NC	LPC500	LPC750	SEM	*p*-value
1 ~ 21 days (g)						
Final weight	842.8^ab^	839.9^b^	849.6^ab^	851.1^a^	3.76	<0.01
ADG	38.04^b^	37.98^b^	38.39^ab^	38.56^a^	0.181	<0.01
ADFI	48.13^ab^	48.07^b^	49.09^a^	49.29^a^	0.229	<0.01
F/G ration	1.27	1.27	1.28	1.28	0.008	0.480
22 ~ 42 days (g)						
Final weight	2,640.2^a^	2629.6^b^	2712.2^a^	2757.8^a^	20.80	<0.01
ADG	85.59^a^	85.22^b^	88.70^a^	90.79^a^	0.992	<0.01
ADFI	144.83	147.48	145.28	144.07	1.271	0.489
F/G ratio	1.69^a^	1.73^a^	1.64^b^	1.59^c^	0.015	<0.01
1 ~ 42 days (g)						
Final weight	2640.2^a^	2629.6^b^	2712.2^a^	2757.8^a^	20.80	<0.01
ADG	61.82^a^	61.60^b^	63.54^a^	64.67^a^	0.495	<0.01
ADFI	95.30	96.56	96.01	95.52	0.666	0.848
F/G ratio	1.54^ab^	1.57^a^	1.51^b^	1.48^b^	0.010	<0.01

^a–d^Means with different letters in each row were significantly different at *P* < 0.05. ADG, average daily gain; ADFI, average daily feed intake; F/G, feed to gain. CON, basal diet; NC, basal diet with energy reduced by 60 kcal/kg; LPC500, NC diet + 500 mg/kg LPC; LPC750, NC diet + 750 mg/kg LPC. SEM, standard error of the mean (*n* = 6).

At 22–42 days, both the 500 and 750 mg/kg LPC supplements significantly reduced the F/G ratio compared with the NC and CON groups (*p* < 0.05). Notably, the 750 mg/kg LPC supplementation led to a significantly lower F/G ratio than both control groups (*p* < 0.05).

At 1–42 days, compared with the NC group, LPC at 500 and 750 mg/kg increased final weight and ADG while decreasing F/G ratio (*p* < 0.05).

### 3.2 Apparent nutrient digestibility

As shown in Table 3, supplementation with 750 mg/kg LPC significantly increased EE digestibility compared with the NC and CON groups and increased OM digestibility compared with the NC group at 21 days (*p* < 0.05). Moreover, at 21d, AME of LPC supplementation was significantly higher compared with NC group especially, 750 mg/kg LPC supplementation also significantly increased AME compared with other groups (*p* < 0.05). At 42 days, supplementation with 750 mg/kg LPC significantly increased the digestibility of DM, OM, CP, and EE compared with the NC group (*p* < 0.05); the increases in OM and EE were also significant when compared with the CON group (*p* < 0.05). Additionally, there was a significant increase in EE digestibility in the LPC500 group compared with the CON and NC groups at 42 days (*p* < 0.05).

**Table 3:** Effects of LPC supplementation on apparent nutrient digestibility in broilers (%).

Items	CON	NC	LPC500	LPC750	SEM	*p*-value
21 days						
DM	75.88	75.65	76.12	76.09	0.161	0.322
OM	78.49^ab^	78.29^b^	78.92^ab^	79.12^a^	0.001	0.025
CP	66.69	66.61	66.60	67.29	0.338	0.057
EE	52.89^b^	52.55^b^	54.37^ab^	56.02^a^	0.610	<0.01
AME MJ/kg	15.64^bc^	15.61^c^	15.71^b^	15.72^a^	0.008	<0.01
42 days						
DM	73.13^ab^	72.09^b^	72.09^ab^	75.08^a^	0.420	<0.01
OM	74.63^b^	74.23^b^	75.93^ab^	76.33^a^	0.376	<0.01
CP	52.34^ab^	50.25^b^	53.18^ab^	55.09^a^	0.637	<0.01
EE	75.22^c^	81.12^b^	84.80^a^	85.88^a^	0.691	<0.01
AME MJ/kg	16.58^a^	16.42^b^	16.49^ab^	16.46^ab^	0.029	<0.01

^a–d^Means with different letters in each row were significantly different at *P* < 0.05. DM, dry matter; OM, organic matter; CP, crude protein; EE, ether extract. CON, basal diet; NC, basal diet with energy reduced by 60 kcal/kg; LPC500, NC diet + 500 mg/kg LPC; LPC750, NC diet + 750 mg/kg LPC. SEM, standard error of the mean (*n* = 6).

### 3.3 Carcass traits

As listed in Table 4, at 21 days, compared with the CON and NC groups, LPC addition significantly reduced the percentage of abdominal fat and increased thigh muscle yield; especially, the 750 mg/kg LPC group significantly increased the dressing percentage (*p* < 0.05). Moreover, the percentages of eviscerated yield, breast muscle, and thigh muscle showed a marked increase in the 750 mg/kg LPC group compared with those in the NC group (*p* < 0.05). At 42 days, supplementation with 750 mg/kg LPC significantly increased the dressing percentage and breast muscle rate compared with the NC group (*p* < 0.05), while decreasing the abdominal fat rate and increasing the breast muscle yield compared with the CON group (*p* < 0.05).

**Table 4:** Effects of LPC supplementation on carcass traits in broilers (%).

Items	CON	NC	LPC500	LPC750	SEM	*p*-value
21 days						
Semi-eviscerated yield	87	87	87	87	0.143	0.702
Eviscerated yield	73^ab^	72^b^	73^ab^	73^a^	0.174	<0.01
Dressing percentage	93^b^	93^b^	93^b^	94^a^	0.138	0.003
Breast muscle yield	25^a^	24^b^	25^a^	25^a^	0.002	<0.01
Thigh muscle yield	16^b^	16^b^	17^a^	17^a^	0.001	<0.01
Percentage of abdominal fat	1.6^a^	1.6^a^	1.4^b^	1.3^b^	0.005	<0.01
42 days						
Semi-eviscerated yield	83	83	83	83	0.146	0.676
Eviscerated yield	72^a^	71^b^	73^a^	73^a^	0.031	0.019
Dressing percentage	88^ab^	87^b^	88^ab^	89^a^	0.003	<0.01
Breast muscle yield	29^b^	29^b^	29^b^	30^a^	0.002	<0.01
Thigh muscle yield	17	17	17	18	0.002	0.609
Percentage of abdominal fat	1.46^a^	1.30^b^	1.40^ab^	1.31^b^	0.036	0.014

^a–d^Means with different letters in each row were significantly different at *P* < 0.05. CON, basal diet; NC, basal diet with energy reduced by 60 kcal/kg; LPC500, NC diet + 500 mg/kg LPC; LPC750, NC diet + 750 mg/kg LPC. SEM, standard error of the mean (*n* = 6).

### 3.4 Serum biochemistry indices

The effects of LPC supplementation on the serum biochemistry in broilers are indicated in Table 5. The ALB content in the LPC750 group was higher than that in other groups (*p* < 0.05). In addition, dietary LPC supplementation significantly decreased UREA and TG content, and LDH activity compared with the CON group (*p* < 0.05), and decreased ALT activity compared with the CON and NC groups (*p* < 0.05). The TC content and AST activity decreased significantly in the LPC750 group compared with the CON group (*p* < 0.05). The MDA content in the LPC750 groups was lower than that in the NC and LPC500 groups (*p* < 0.05). The contents of UREA, and activities of ALT and LDH were significantly decreased in the NC group compared with those in the CON group (*p* < 0.05).

**Table 5:** Effects of LPC supplementation on the serum biochemistry in broilers.

Items	CON	NC	LPC500	LPC750	SEM	*p*-value
GLU, mmol/L	11.02	9.27	9.28	9.21	0.261	0.05
TP, g/L	31.12	31.55	32.62	31.22	0.416	0.796
ALB, g/L	7.25^b^	7.23^b^	7.32^b^	7.98^a^	0.131	0.013
UREA, mmol/L	2.01^a^	1.71^b^	1.73^b^	1.70^b^	0.032	<0.01
TG, mmol/L	0.65^a^	0.59^ab^	0.54^b^	0.51^b^	0.016	0.002
TC, mmol/L	3.18^a^	3.02^ab^	2.64^ab^	2.46^b^	0.089	<0.05
HDL, mmol/L	1.53	1.54	1.57	1.69	0.032	0.117
LDL, mmol/L	0.69	0.69	0.60	0.56	0.020	0.05
ALT, U/mL	16.00^a^	13.33^b^	9.17^c^	9.67^c^	0.484	<0.01
AST, U/mL	640^a^	481^ab^	473^ab^	405^b^	24.4	<0.05
ALP, U/mL	1,146	1,407	1,301	1,295	58.6	0.802
LDH, U/mL	3,369^a^	2,496^b^	2,361^b^	2,385^b^	112.5	<0.01
MDA, nmol/mL	3.58^b^	4.38^ab^	4.76^a^	3.13^b^	0.179	<0.01

^a–d^Means with different letters in each row were significantly different at *P* < 0.05. GLU, glucose; TP, total protein; ALB, albumin; UREA, urea; TG, triglycerides; TC, total cholesterol; LDL, low-density lipoprotein; HDL, high-density lipoprotein; ALT, alanine aminotransferase; AST, aspartate aminotransferase; ALP, alkaline phosphatase; LDH, lactate dehydrogenase; MDA, malondialdehyde. CON, basal diet; NC, basal diet with energy reduced by 60 kcal/kg; LPC500, NC diet + 500 mg/kg LPC; LPC750, NC diet + 750 mg/kg LPC. SEM, standard error of the mean (*n* = 6).

### 3.5 Meat quality

As shown in Table 6, compared with the CON and NC groups, the intramuscular fat of breast muscle increased significantly in the LPC groups (*p* < 0.05). In addition, in the breast muscle, supplementation with 500 mg/kg LPC decreased the drip loss and b* compared with the NC group (*p* < 0.05). The value of L* was decreased in the LPC750 group compared with the control group (*p* < 0.05), while the a* value was increased compared with the NC group (*p* < 0.05). In the thigh muscle, LPC supplementation increased intramuscular fat compared with the NC group (*p* < 0.05). Drip loss in the LPC500 group was lower than that in the CON and NC groups (*p* < 0.05). In addition, compared with the NC group, supplementation with 750 mg/kg LPC decreased b* value (*p* < 0.05).

**Table 6:** Effects of LPC supplementation on meat quality in broilers.

Items	CON	NC	LPC500	LPC750	SEM	*p*-value
Breast muscle						
Intramuscular fat, %	3.04^b^	2.79^b^	3.41^a^	3.45^a^	0.086	<0.01
Drip loss, %	4.71^ab^	5.00^a^	4.39^b^	4.41^ab^	0.144	0.030
Cooking loss, %	22.36	22.42	22.32	22.19	0.249	0.326
Shear force, N	41.39	40.91	41.59	42.50	0.588	0.162
L*	50.94^a^	50.09^ab^	49.46^ab^	49.05^b^	0.420	<0.01
a*	1.88^ab^	1.64^b^	1.83^ab^	2.19^a^	0.131	<0.01
b*	10.56^a^	10.61^a^	9.50^b^	10.55^a^	0.237	<0.01
Thigh muscle						
Intramuscular fat, %	8.48^a^	7.42^b^	8.45^a^	8.64^a^	0.222	<0.01
Drip loss, %	3.53^a^	3.24^a^	2.24^b^	2.79^ab^	0.166	0.009
Cooking loss, %	17.60	18.13	17.58	17.51	0.298	0.078
Shear force, N	24.24	24.13	24.50	24.73	0.798	0.175
L*	55.09	54.55	54.71	52.93	0.578	0.19
a*	6.95	6.29	6.15	6.36	0.384	0.104
b*	10.56^b^	12.39^a^	11.70^ab^	10.34^b^	0.371	<0.01

^a–d^Means with different letters in each row were significantly different at *P* < 0.05. L*: lightness; a*: redness; b*: yellowness. CON, basal diet; NC, basal diet with energy reduced by 60 kcal/kg; LPC500, NC diet + 500 mg/kg LPC; LPC750, NC diet + 750 mg/kg LPC. SEM, standard error of the mean (*n* = 6).

### 3.6 Hepatic lipid metabolism

As shown in Table 7, at 21 days of age, TC, TG, and MDA levels were significantly lower in the NC group (*p* < 0.05), and TG and MDA levels were significantly lower in the LPC groups compared with the CON group (*p* < 0.05). Compared with the NC group, TG level was significantly higher (*p* < 0.05) in the LPC500 group, while it was significantly lower in the LPC750 group (*p* < 0.05). At 42 days of age, compared with the CON group, TG level was significantly lower in the LPC groups (*p* < 0.05), and MDA levels were significantly lower in the LPC750 group (*p* < 0.05). Compared with the NC group, the 500 and 750 mg/kg LPC groups had significantly higher TC levels in other groups (*p* < 0.05).

**Table 7:** Effects of LPC supplementation on the hepatic lipid metabolism in broilers.

Items	CON	NC	500	750	SEM	*p*-value
21 days						
TC, mmol/L	2.95^a^	2.72^b^	2.82^ab^	2.65^b^	0.033	<0.01
TG, mmol/L	0.57^a^	0.39^c^	0.46^b^	0.35^d^	0.015	<0.01
MDA, μmol/L	3.69^a^	3.37^b^	2.52^d^	2.81^c^	0.118	<0.01
42 days						
TC, mmol/L	0.12^a^	0.08^b^	0.11^a^	0.11^a^	0.003	<0.01
TG, mmol/L	0.58^a^	0.53^ab^	0.42^c^	0.51^b^	0.013	<0.01
MDA, μmol/L	3.82^a^	3.76^ab^	3.60^ab^	3.39^b^	0.02	<0.01

^a–d^Means with different letters in each row were significantly different at *P* < 0.05. TC, total cholesterol; TG, triglycerides; MDA, malondialdehyde. CON, basal diet; NC, basal diet with energy reduced by 60 kcal/kg; LPC500, NC diet + 500 mg/kg LPC; LPC750, NC diet + 750 mg/kg LPC. SEM, standard error of the mean (*n* = 6).

## 4 Discussion

In this study, dietary LPC supplementation improved broiler growth performance, with 750 mg/kg LPC showing the greatest effect. Previous studies have shown that broiler ADG decreases when dietary energy levels are reduced (30, 31). In the present study, the ADG was increased with the addition of LPC to a low-energy diet, which suggests that dietary addition of LPC could improve broiler growth. Similarly, a study demonstrated that LPC supplementation increased body weight gain in broilers during 1–10, 11–24, and 1–35 days of age (32). In addition, it was found that 750 mg/kg LPC significantly decreased the F/G ratio when LPC was added at 1–42 days in the current study. In parallel, a previous study showed that LPC supplementation presented a significantly lower F/G ratio (33). However, it has been reported that 0.04% lysolecithin supplementation in a low-energy diet (metabolizable energy is 2,800 kcal/kg) did not affect the F/G ratio of broilers in the overall experimental period (34); the results differed from those of the present study, probably due to differences in diet composition or biosurfactants.

In the present study, it was found that the addition of LPC in a low-energy level diet increased the apparent nutrient digestibility of nutrients in broilers, especially EE. The result was consistent with the previous finding that dietary supplementation with LPC significantly increased the metabolizable energy and EE digestibility in broilers compared with the control group (35). Similarly, it has been shown that dietary LPC addition significantly improved the digestibility of DM and EE in broilers (36). In addition, the study found that LPC supplementation for the low-energy group resulted in significantly better digestibility of CP and EE than the low-energy group (37). Furthermore, similar to the present study, it found that the broilers fed with higher levels of LPC (200–400 g/100 kg) were superior to lower levels (100 g/100 kg) in improving the metabolism of nutrients (38). These suggest that LPC could improve nutrient absorption in the broiler intestine; the physiological mechanism underlying these consistent improvements primarily stems from LPC’s role as a potent emulsifier.

In livestock production, slaughter performance served as a critical economic indicator, directly reflecting nutrient deposition and meat yield. Excessive abdominal fat deposition not only compromised meat flavor but also diminished overall carcass quality (39, 40). Research indicated that supplementing low-energy diets with 750 mg/kg LPC significantly increased dressing percentage and breast meat yield while markedly reducing abdominal fat percentage compared with controls. This effect likely arose from LPC enhancing fat digestion and increasing the efficiency of fat absorption, thereby redirecting more dietary energy and nutrients toward muscle protein synthesis rather than adipose tissue deposition. The positive impact of LPC on slaughter performance in low-energy diets aligned with prior research. For instance, combining LPC with other emulsifiers in low-energy diets similarly enhanced carcass yield (41); another study demonstrated that 0.075% LPC (equivalent to 750 mg/kg) improved slaughter yield in broilers (42). However, adding emulsifiers to normal-energy diets did not significantly improve slaughter performance (33). We hypothesized that in normal-energy diets, endogenous bile secretion may already sufficiently emulsify fats, making it difficult for exogenous emulsifiers to exert significant effects. In low-energy diets, adding LPC was crucial to compensate for dietary energy deficiency and to maximize fat utilization. Thus, differences in research outcomes may have stemmed not only from the emulsifier’s composition or dosage, but also from whether dietary conditions created metabolic demands that emulsifiers could effectively address.

Serum biochemical markers served as key indicators of systemic nutritional metabolism and health status, reflecting the complex interplay between dietary patterns, nutritional levels, and physiological stages (43). Under low-energy dietary conditions, this study found that LPC supplementation significantly reduced serum TG, TC, MDA, ALT, and AST levels, a particularly noteworthy finding. The reduction in triglycerides and total cholesterol indicated a fundamental improvement in systemic lipid metabolism (7, 15, 44). This effect may have stemmed from LPC’s role as a potent emulsifier, enhancing the efficiency of intestinal fat digestion and absorption. The significant decrease in MDA, a marker of lipid peroxidation (45), suggested alleviated oxidative stress. This suggested that LPC not only influenced lipid digestion but also exerted antioxidant effects, potentially by reducing substrates for peroxidation reactions or enhancing overall antioxidant capacity in broilers. Reduced ALT and AST enzyme activities were observed in the low-energy diet model of broilers. ALT and AST enzymes are released into the bloodstream when hepatocytes are damaged (46). However, studies had reported that LPC had no significant effect on these enzymes in broilers under different dietary conditions (38). Under the metabolic stress of a low-energy diet, when the liver was stressed, the positive impact of LPC on hepatocyte integrity became more pronounced and readily observable. This was speculated to result from improvements in systemic lipid profiles and oxidative stress.

Meat quality parameters in livestock and poultry served as comprehensive indicators reflecting muscle biochemical and structural characteristics, directly determining eating experience and commercial value (47, 48). This study found that supplementing low-energy diets with dietary LPC significantly improved broiler meat quality, including reduced drip loss, optimized meat color, and enhanced intramuscular fat (IMF) deposition. Studies have reported that meat with higher drip loss exhibited greater acidity, lower protein content, poorer sensory quality, and elevated lipid oxidation (49, 50). The observed reduction in drip loss aligned with previous findings in broilers fed LPC-supplemented low-energy diets (51). This effect may have resulted from LPC promoting a more stable post-slaughter muscle energy metabolism, thereby enhancing meat’s water retention capacity. Broiler meat color was a critical quality indicator, with ideal color reflecting good myoglobin oxygenation and low lipid oxidation (52). The significant improvement in color parameters observed with LPC supplementation in this study indicated enhanced myoglobin oxygenation capacity and reduced lipid oxidation. This aligned with previous findings that emulsifier supplementation in low-energy diets improved breast meat color (53). The mechanism may have involved LPC-enhanced antioxidant status, delaying myoglobin and lipid oxidation, thereby preserving bright red color. Notably, the 500 mg/kg LPC supplement significantly promoted IMF deposition. As a key reservoir of flavor precursors, IMF showed a positive correlation with meat juiciness and aroma (54). It was hypothesized that LPC may have promoted the synthesis and deposition of triacylglycerol in the IMF reservoir by influencing lipid metabolism within adipocytes or muscle fibers.

In broilers, a significant proportion of lipids deposited in adipose tissue are synthesized *de novo* in the liver, which is the central organ for lipid and energy intermediary metabolism (55). Excessive dietary energy and fat intake in broilers elevate serum and hepatic TG and TC levels, promoting ectopic lipid deposition in the liver, and this lipid accumulation triggers lipotoxicity, characterized by enhanced lipid peroxidation, oxidative stress, and pro-inflammatory responses (56, 57). Consequently, hepatic MDA content not only reflects the extent of lipid peroxidation mediated by oxygen-free radicals but also indirectly indicates the degree of oxidative injury (58, 59). Multiple experimental datasets indicated that dietary emulsifier supplementation significantly enhanced hepatic lipase activity in broilers, improved lipid digestibility, and reduced TG, TC, and MDA concentrations (36, 60, 61). In parallel with emulsifiers, LPC supplementation decreased the expression of the hepatic lipogenic genes (44, 53). Consistently, our study showed that LPC supplementation effectively reduced hepatic TG and MDA levels, with the 750 mg/kg LPC treatment demonstrating superior regulatory efficacy.

## 5 Conclusion

This study demonstrates that supplementing low-energy diets with LPC significantly improves broiler growth performance (e.g., ADG, F/G ratio), enhances apparent nutrient digestibility, optimizes slaughter performance and meat quality (e.g., drip loss and meat color), and improves hepatic lipid metabolism (evidenced by reduced TC, TG, and MDA). These improvements are primarily attributed to LPC-promoted fat digestibility, enabling more efficient energy utilization in low-cost, low-energy diets and thereby enhancing overall feeding economy. Additionally, improved nutrient (particularly lipid) utilization efficiency positively impacts the environment by reducing nutrient excretion. However, the precise mechanisms by which LPC modulates the expression of genes related to hepatic lipid metabolism remain incompletely understood. Therefore, further molecular analyses are required to elucidate its potential regulatory pathways.

## Data Availability

The original contributions presented in the study are included in the article/supplementary material, further inquiries can be directed to the corresponding author.
